# Don’t Monkey with the Buz-Saw

**Published:** 1896-11

**Authors:** 


DON'T MONKEY WITH THE BUZ-SAW.—Our esteemed young contemporary at Houston is not to be sneezed at. She's little, but she's loud. With the characteristic pluck and bluster of the bantam species, she comes back at us in gallant style, giving us, not a Roland for an Oliver, but a Red for a Red Back, hitting us *real hard*. We are given to understand that if the Red Back *is* a whale, the *S. W. Medical Record* is no sardine; and when we are bragging on the TEXAS MEDICAL JOURNAL we are respectfully requested to remember that "there are others."

We didn't know 'twas loaded. Won't somebody kindly help us let the *Record* alone?

Seriously, in our strictures on the *Record* we have seemingly been unkind; but only seemingly. What we have written was intended for good natured banter,—criticism, for the most part, of misspelled words, which we knew well enough, might have been, and most likely were, typographical errors, or the fault of the proof reader in some way, something we are all liable to, and guilty of;--because the owners and editors of the *Record*, so far as known to us, are educated gentlemen, and physicians of repute. Our personal acquaintance extends to only two of them, Drs. Red and Morris, and of those two, one is the writer's warm, personal friend, and we wish to assure them that we have no intention or desire to reflect, in any way, upon any of them; and it is far from us to seek to injure or detract from the merits of their publication. Everything must have a start, and time was when the *Red Back* was small and young (but, always, like a bantam, it was ready to fight). We doubt the propriety of the endeavor to establish another medical journal in Texas at this time, where there are already more than enough; and question the success of the venture. Still, we are not a bit jealous, and we wish it well, and will be glad to see it succeed. It is meritorious, and the management have our respect and good will. We will be pleased, really, to help them secure subscribers, or to aid them in any way in our power,—like an older brother, who remembers that he was once a kid himself.

